# Evolving Deep Architecture Generation with Residual Connections for Image Classification Using Particle Swarm Optimization

**DOI:** 10.3390/s21237936

**Published:** 2021-11-28

**Authors:** Tom Lawrence, Li Zhang, Kay Rogage, Chee Peng Lim

**Affiliations:** 1 Department of Computer and Information Sciences, Faculty of Engineering and Environment, Northumbria University, Newcastle upon Tyne NE1 8ST, UK; tom.lawrence@northumbria.ac.uk (T.L.); k.rogage@northumbria.ac.uk (K.R.); 2 Department of Computer Science, Royal Holloway, University of London, Egham TW20 0EX, UK; 3 Institute for Intelligent Systems Research and Innovation, Deakin University, Waurn Ponds, VIC 3216, Australia; chee.lim@deakin.edu.au

**Keywords:** deep architecture generation, deep residual network, particle swarm optimization, image classification

## Abstract

Automated deep neural architecture generation has gained increasing attention. However, exiting studies either optimize important design choices, without taking advantage of modern strategies such as residual/dense connections, or they optimize residual/dense networks but reduce search space by eliminating fine-grained network setting choices. To address the aforementioned weaknesses, we propose a novel particle swarm optimization (PSO)-based deep architecture generation algorithm, to devise deep networks with residual connections, whilst performing a thorough search which optimizes important design choices. A PSO variant is proposed which incorporates a new encoding scheme and a new search mechanism guided by non-uniformly randomly selected neighboring and global promising solutions for the search of optimal architectures. Specifically, the proposed encoding scheme is able to describe convolutional neural network architecture configurations with residual connections. Evaluated using benchmark datasets, the proposed model outperforms existing state-of-the-art methods for architecture generation. Owing to the guidance of diverse non-uniformly selected neighboring promising solutions in combination with the swarm leader at fine-grained and global levels, the proposed model produces a rich assortment of residual architectures with great diversity. Our devised networks show better capabilities in tackling vanishing gradients with up to 4.34% improvement of mean accuracy in comparison with those of existing studies.

## 1. Introduction

Automatically constructing convolutional neural networks (CNNs) is a challenging task, since handcrafting CNNs requires expert knowledge and significant manual trial-and-error effort. When designing a deep network architecture, design choices include selecting a suitable kernel size owing to its importance in adjusting the receptive field [1]. Other important steps include balancing the depth of the network by adding or removing layers, as well as increasing or decreasing the number of filters per layer within the network [2].

Popular CNN architectures such as LeNet [3], ResNet [4], Wide ResNet [2] and DenseNet [5] have been designed by hand to compete on benchmark datasets such as ImageNet [6], MS COCO [7], CIFAR-10 and CIFAR-100 [8]. Such network architectures have been hand-crafted by domain experts in their respective fields. These hand-crafted architectures and concepts introduced are often used as a starting point when designing a new architecture from scratch, with respect to tasks in new domains. As an example, ref. [9] used a DenseNet-inspired network to perform keyword spotting on smart terminal devices, while the ResNet architecture was adopted by [10] for medical diagnosis.

The disadvantage of using an existing, state-of-the-art but hand-crafted network as a starting point is that the network is often overly large for the task at hand. This is because the design choices made during the construction of the hand-crafted networks normally aim to maximize accuracy on challenging competition datasets. Such state-of-the-art deep networks require a substantial amount of training data compared with those used for training shallower networks [11]. When tackling new and novel image classification problems, it is usually a challenging task to obtain a sufficient amount of data. To combat this, methods such as pruning convolutional kernels have been proposed, to slim down networks by up to 10 times smaller [12]. This is measured as a reduction in the convolutional kernels of the final network architecture. A weakness of the pruning approach is that it requires a suitable network as the starting point. On the other hand, transfer learning is another mechanism used to leverage state-of-the-art CNN architectures in new application domains. Many existing studies employ transfer learning to fine-tune a pre-trained network for undertaking a task in a new domain. As an example, a pre-trained ResNet network was re-trained by [13] for tackling malicious software classification problems. However, such a transfer learning process relies heavily on existing hand-crafted architectures. Since these state-of-the-art base networks [2,4,5] are often originally constructed for large and complex datasets (such as ImageNet [6] and MS COCO [7]) and the new domain is often a narrower problem (e.g., number plate recognition), the adopted models could be overly complex. Moreover, in some cases, if a new domain is very different from the pre-trained domain, the prior knowledge may not be relevant or sufficient enough, which may constrain model performance.

Owing to the aforementioned weaknesses and challenges, a new research era emerges which aims to address the research question of how to automatically design deep CNN architectures for tackling problems in new domains. Such research efforts have resulted in some impressive developments in the field where evolutionary algorithms are used to automatically devise CNN models from scratch. As an example, sosCNN [14], psoCNN [15] and GeNET [16], employed particle swarm optimization (PSO) [17] and generic algorithms (GAs), respectively, for devising deep networks.

### 1.1. Research Problems

The weaknesses of current state-of-the-art research on CNN architecture generation are as follows. On one hand, existing automated methods purely optimize important design choices such as network depths, kernel sizes and pooling types. Moreover, they do not take advantage of modern strategies such as skip or dense connections. This leads to undesirable effects, such as the vanishing gradient problem [4] and underfitting [18] as depths increase. On the other hand, existing methods which take skip or dense connections into account, such as [5,19,20], limit the search spaces by fixing the kernel sizes, pooling types and numbers of blocks. They purely focus on optimizing the optimal number of layers within a block and model width. This compromises the variety of the generated networks.

Therefore, a gap in existing knowledge is identified, i.e., research on a new architecture generation method to automatically construct a deep CNN network architecture, which not only exploits modern strategies such as skip connections, but also remains capable of constructing new and novel network topologies with a high degree of diversity. To address this gap in knowledge, we propose a novel PSO variant by incorporating a new encoding strategy and a new velocity updating mechanism capable of constructing deep CNN architectures comprising skip connections. Our approach maintains the ability to devise diverse CNN architectures by automatically formulating network architectures composed of different kernel sizes, pooling types, depths and widths.

**Figure 1:**
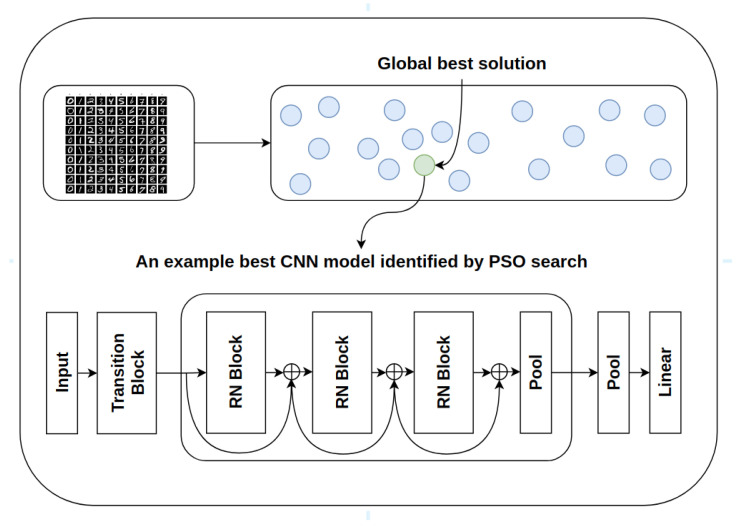
The proposed system architecture where the identified best residual network architecture is indicated by the global best solution.

In addition, most existing search methods, such as sosCNN [14] and psoCNN [15], adopt the traditional PSO operations by using the personal and global best solutions to guide the search process. Thus, their search processes are more likely to be trapped in local optima, owing to the dominance of a single global best leader. Besides that, the architecture search is a challenging multimodal optimization problem where there are multiple local optimum solutions present in the search space. We propose a PSO variant with multiple leaders, i.e., the neighboring best solutions as well as the swarm leader, for deep architecture generation, to overcome limitations (e.g., local optimum traps) of existing search methods and address the gap in knowledge. Figure 1 illustrates the system architecture.

In summary, there are two key differences between the proposed PSO model in this research study and those in the aforementioned closely related studies, e.g., the original PSO algorithm, psoCNN [15] and sosCNN [14]. The key differences are the following: (1) our encoding strategy considers skip connections, whereby related studies do not exploit skip connections; (2) our search strategy is guided by the neighboring and global best solutions, whereby related studies (e.g., PSO, psoCNN [15] and sosCNN [14]) are guided by personal and global best solutions.

### 1.2. Contributions

Specifically, the contributions of this research study are as follows:

- A new PSO algorithm, namely resPsoCnn, is proposed for residual deep architecture generation. The novel aspects of the resPsoCnn model include (1) a new residual group-based encoding scheme and (2) a new search mechanism guided by neighboring and global promising solutions for deep architecture search. Specifically, the new group-based encoding scheme is able to describe network configurations with residual connections. In the encoding scheme, candidate models are firstly converted into groups. Each group contains one or more convolutional blocks and an optional pooling layer. The number of filters in the convolutional layers in each group, which controls the network width, is also optimized. The kernel sizes of convolutional layers are individually encoded, giving fine-grained control over the receptive field of each block. The number of blocks within each group can vary to increase or decrease the model depth, while different pooling layer types are embedded to control downsampling.
- We propose an optimization strategy that exploits the advantages of skip connections to avoid the vanishing gradient problem. Such a strategy addresses the weaknesses in related studies. As an example, (1) existing studies either perform optimization tasks only on fixed skeleton models (e.g., fixed numbers of blocks with fixed kernel sizes) that exploit skip connections but restrict model diversity, (2) or they optimize a range of hyperparameter settings, capable of producing diverse but shallow networks, without residual connections. Our proposed strategy undertakes both weaknesses by providing the ability to leverage skip connections in establishing deep network architectures, whilst optimizing a range of network settings to improve diversity.
- We propose a new velocity updating mechanism that adds randomness to the updating of both the group and block hyperparameters. Specifically, it employs multiple elite signals, i.e., the swarm leader and the non-uniformly randomly selected neighboring best solutions, for searching optimal hyperparameters. Such a search process guided by multiple promising signals escalates social communication and is more likely to overcome stagnation. The hyperparameter updating procedure at the group and block levels is conducted by either selecting from the difference between the current particle and global best solution, or the difference between the current particle and a neighboring best solution, to increase search diversity. The proposed search mechanism optimizes the number of groups, network width and depth, kernel sizes and pooling layer choices to produce a rich assortment of optimal residual deep architectures. Owing to the guidance of multiple elite signals, our search process achieves a better balance between exploration and exploitation to overcome weaknesses such as the local optimum traps of existing search methods led by only single leader. Evaluated using a number of benchmark datasets, our devised networks produce superior performances in respect to those yielded by several state-of-the-art existing methods.

The paper is organized as follows. We present related studies on deep architecture generation and optimal hyperparameter selection using PSO and other search methods in Section 2. The proposed PSO variant with new encoding and search strategies for deep architecture generation is introduced in Section 3. Section 4 provides a comprehensive evaluation of the proposed model against several state-of-the-art methods using a number of benchmark datasets. We conclude this research study and identify future directions in Section 5.

## 2. Related Studies

In this section, we discuss state-of-the-art related studies on deep architecture generation and hyperparameter fine-tuning using PSO and its variant methods, as well as other evolutionary algorithms.

### 2.1. Deep Architecture Generation Using PSO Methods

The PSO algorithm [17] is a popular swarm intelligence algorithm, which simulates fish schooling and bird flocking. A particle is represented by a position in a multi-dimensional search space. Each element of the particle represents a particular hyperparameter to be optimized. The objective of the PSO algorithm is to find the most optimal position within a search space. The fitness of a particle is measured by an objective function. The search process aims to minimize this cost function.

A swarm is formed by multiple particles. Over a series of iterations, each particle attempts to improve its current position by moving to a new location. The direction and distance of the movement are guided by two elements, i.e., the swarm’s best known position, referred to as the global best, and the particle’s best known position, referred to as the personal best. Equation (1) defines the velocity calculation to determine the direction and scale for position updating for a particular particle during the search process. In Equation (1), $V_{i}^{t}$ and $V_{i}^{t+1}$ represent the velocities for the *i*th particle in the *t*th and t+1th iterations, respectively. $X_{i}^{t}$ and $X_{i}^{t+1}$ denote the positions of the *i*th particle, for the *t*th and t+1th iterations, respectively. $P_{i}^{t}$ represents the personal best position of particle *i* for the *t*th iteration, while $G^{t}$ indicates the global best position for the *t*th iteration. Parameters c1 and c2 are acceleration coefficients, which are typically assigned between 0.5 and 2.5. When c1 is higher than c2, the velocity update is weighted more towards a global search than a local search and vice versa. Parameters r1 and r2 are randomly generated values between 0 and 1, while *w* is a weighting factor to determine the effects of the previous velocity on the generation of new velocity.

(1) $$V_{i}^{t+1}=wV_{i}^{t}+c_{1}r_{1}\left(P_{i}^{t}-X_{i}^{t}\right)+c_{2}r_{2}\left(G^{t}-X_{i}^{t}\right)$$

The new position of particle *i* in the t+1th iteration, i.e., $X_{i}^{t+1}$, is produced using Equation (2), based on the velocity yielded by Equation (1).

(2) $$X_{i}^{t+1}=X_{i}^{t}+V_{i}^{t+1}$$

The PSO algorithm has been widely adopted for automatic CNN architecture generation in related studies, e.g., IPPSO [21], psoCNN [15] and sosCNN [14]. PSO has also been applied to the optimization of hand-crafted models such as DenseNet [5]. As an example, Wang et al. [22] proposed a multi-objective PSO method for DenseNet architecture generation, which maximized accuracy whilst minimizing the computational cost of the devised CNN model. Their PSO operation was used for optimizing the number of dense blocks, layers per block and the growth rate of each block. The architecture search required a high computational cost, i.e., 3 days with the settings of 8 GPUs and a population size of 20, optimized over 20 iterations. Their optimized DenseNet model was evaluated on the CIFAR-10 dataset and achieved an accuracy rate of 95.51%, with an improvement of 0.74% over that of DenseNet-121. However, their work did not optimize other key parameters such as kernel size and pooling types within each dense block.

Dutta et al. [23] proposed two PSO variants, namely qubit fractional order PSO (Qubit FO-DPSO) and qutrit fractional order PSO (Qutrit FO-DPSO). Their PSO methods were used to optimize the wavelength thresholds to minimize signal noise for hyperspectral image (HSI) segmentation. First of all, improved subspace decomposition algorithm, principal component analysis (PCA) and a band-selection CNN were used to conduct discriminative band selection. In addition, their models maintained multiple swarms simultaneously while using quantum parallelism to reduce computational costs. In the Qubit configuration, each dimension was initialized randomly with a binary value of 0 or 1, whereas, in the Qutrit configuration, each dimension was initialized randomly with either 0, 1 or 2. Fractional order (FO) was proposed for velocity calculation. It took the last three velocities of each particle into account and employed a weighted sum of these recent velocities for calculating the new one in the next iteration. Each particle was evaluated using three objective functions, i.e., modified Otsu criterion, Masi entropy and Tsallis entropy, for thresholding performance measurement. Moreover, a quantum disaster operation (denoted as D) was proposed in the aforementioned variants to mitigate early stagnation and increase search diversity. This operator deleted particles and even a whole swarm when the fitness scores did not improve over 10 consecutive iterations. It also generated new particles or a new swarm when a particular swarm illustrated enhanced fitness over generations. Evaluated using benchmark datasets, their models achieved measurable improvements in terms of the peak signal-to-noise ratios and Dice similarity scores in comparison with those of other search methods for HSI segmentation.

Fielding and Zhang [19] proposed a PSO-based method for optimizing skeleton block-based CNN architectures comprising dense connectivity [5]. Their work employed novel weight inheritance learning mechanisms with the attempt to reduce the computational costs. Specifically, their work initialized a network containing four dense blocks. These dense blocks were subsequently optimized using a modified PSO algorithm with cosine search coefficients. The objective was to discover the optimal number of layers within each dense block and the growth rate of the overall model. The growth rate controlled the number of filters within the model, i.e., the model’s width. The weight inheritance processes were subsequently applied to any CNN architecture devised by the PSO variant. They employed the layer position and the size of its parameter matrix as the search key for weight inheritance. Evaluated on the CIFAR-10 dataset, in comparison with related studies such as [24], their method reduced the computational cost of the architecture search from 1000 to 150 GPU hours and also improved the accuracy rate from 89% to 90.28%. Moreover, their model did not optimize lower-level features, such as the kernel sizes, pooling types, or the number of blocks, which could restrict their network diversity.

A PSO variant was proposed by Zhang et al. [25] for optimal hyper-parameter selection for evolving ensemble hybrid network construction pertaining to human action recognition. Each base network within the ensemble model was composed of a GoogLeNet in combination with a bidirectional long short-term memory (BLSTM) network. Hyperparameters, such as the learning and dropout rates and the number of hidden units in the BLSTM layers, were optimized using their proposed PSO variant. The PSO operation was guided by hybrid leader signals generated using nonlinear crossover operators, as well as 3D superellipse coefficients, to overcome stagnation. A number of base googLeNet-BLSTM networks were optimized using their PSO method. The devised base networks were subsequently used to construct ensemble models. Evaluated using several well-known human action datasets (e.g., KTH, UCF50 and UCF101), their ensemble networks showed impressive performance in comparison with those of ensemble models devised by other PSO variants and existing state-of-the-art methods.

Liu et al. [26] developed two PSO-based methods, i.e., PSO-Net and CPSO-Net, for cell-based CNN architecture generation with respect to HSI classification. Their encoding process was used to transform architectures into arrays by embedding information such as connections and operation types between network nodes. In both methods, PSO was used to devise optimal CNN architectures. In particular, in CPSO-Net, a SuperNet was maintained first, which was trained using gradient descent. Each particle subsequently inherited the network weights from those of this fixed SuperNet. In comparison with PSO-Net, where each network devised by each particle was trained individually, the SuperNet in CPSO-Net was trained only once per iteration using the gradients of all particles in the swarm to accelerate optimal network generation process. Evaluated using biased and unbiased HSI datasets, their methods obtained improved accuracy rates as compared with those of existing state-of-the-art studies.

An evolutionary group-based PSO (EGPSO) was proposed by Juang et al. [27] for optimizing weights in recurrent neural networks (RNNs) with respect to the generation of forward walking gait of a hexapod robot. Their model incorporated group-based GAs with PSO, which outperformed other GA and PSO methods for optimizing the walking speed of a robot. Tan et al. [28] proposed a PSO variant for optimal hyperparameter selection for a VGG network for melanoma classification. Their PSO variant employed three subswarms guided by distinctive adaptive nonlinear search coefficients, as well as sub-dimension-based search for leader enhancement. A wrapper-based feature selection was also conducted using the PSO algorithm for ensemble model construction pertaining to lesion classification. Moreover, a PSO method with elliptical search coefficients was proposed by [29] for hyperparameter fine-tuning of a mask R-CNN for medical image segmentation, while PSO, in combination with random walk strategies and FA operations [30], was exploited for K-Means clustering centroid enhancement and deep architecture generation for image segmentation and classification, respectively. PSO-based generative adversarial networks (GANs) were also proposed by [31] for facial image generation. The PSO model was used to optimize the parameters of the generator network to improve training stability. The quality and diversity measurements of generated images were taken into account in the cost function. Evaluated using the CelebA dataset, the PSO-enhanced GAN model outperformed the original GAN and other variant methods and overcame the vanishing gradient problem of the original GAN model.

### 2.2. Deep Architecture Generation Using Other Search Methods

There are other search methods used for CNN architecture generation. The sosCNN [14] method was proposed by [14] for deep network generation, based on the search operations of psoCNN [15]. It employed a symbiotic organisms search (SOS) [32] algorithm for the search of evolving network architectures, by introducing two new strategies. Firstly, since the original SOS algorithm excessively eliminated deep networks early in the search process, a slack gain strategy was proposed for devising architectures with greater depths. Specifically, the difference between the global best position and the current particle position was calculated, then a random number was generated for each layer in the network. If the random number was smaller than 0.5, the difference between the global best and the current position was selected for particle position updating. Otherwise, the original particle position was selected. Secondly, a dissimilar mutation strategy was introduced which strictly limited the difference in mutations. This ensured that, when a block mutation occurred, the resulting block was not too dissimilar. The authors claimed that such a process also helped to ensure faster convergence. The work achieved an error rate of 0.3% on the MNIST dataset. However, both sosCNN and psoCNN [15] do not construct models comprising residual connections; therefore, they are susceptible to the vanishing gradient problem [4,33].

In order to better balance the search between exploration and exploitation of the monarch butterfly optimization (MBO) algorithm [34], a new hybrid variant model, namely, the MBO-artificial bee colony firefly enhanced (MBO-ABCFE) algorithm, was proposed [35] for deep architecture generation. The model incorporated MBO with artificial bee colony (ABC) and firefly algorithm (FA) to increase search diversity and overcome stagnation of the original MBO algorithm [36]. Specifically, it diversified global exploration by incorporating the search mechanisms of ABC [37], along with a control parameter which adjusted the intensification. The local exploitation was also increased by adopting the search strategy of the FA [38]. In addition, two new parameters were introduced, i.e., an exhaustiveness parameter and a trial parameter. After each iteration, if an individual butterfly solution did not improve, the trial parameter of the butterfly was increased by one. Once the trial parameter exceeded the exhaustiveness parameter, a new individual was randomly initialized. Therefore, the search exploration was further improved by replacing poor performing individuals stuck in local optima with new solutions. A number of hyperparameters were optimized in their work, i.e., the number of convolutional layers, kernel size, type of activation functions, pooling size, batch size and learning rate. Evaluated using the MNIST dataset, MBO-ABCFE-devised networks achieved an error rate of 0.34% with an improvement of 0.02% over MBO-optimized models.

Chen et al. [39] proposed a BASCNN method by applying a recently proposed meta-heuristic algorithm, i.e., beetle antenna search (BAS) [40], for optimization of CNN hyper-parameters. The BAS algorithm models food-sensing behaviors of beetles and searches for optimal solution in the search space using a single search agent. It was used to optimize initial weights and biases of a LeNet model at the early stage of model training. The model was evaluated using a brain CT scan dataset containing 200 images, half of which were taken from patients with intracranial hemorrhage. The BASCNN model achieved an accuracy rate of 93.93%, outperforming those of existing studies, such as [41,42]. Moreover, the experiments were limited to optimization of the CNN’s initial weights and biases, without optimizing the network structures.

A GeNET model was proposed in [16] for CNN architecture generation based on the GA method. Their work employed an encoding scheme based on a fixed-length binary string. This fixed-length binary string encoded the inter-connections between CNN architecture nodes. Each node contained a set of convolutional, batch-normalization and ReLU layers. Genetic operations such as selection, mutation and crossover mechanisms were subsequently conducted for architecture search. In particular, the mutation operation operated with a low probability of flipping a bit within the fixed-length binary string, thus slightly altering the node connections. The work achieved error rates of 0.34%, 5.39% and 25.12% for the MNIST, CIFAR-10 and CIFAR-100 datasets, respectively.

Architecture generation has also been investigated by combining strategies from multiple evolutionary techniques simultaneously. As an example, Tirumala [43] proposed a multi-population competitive and cooperative neuroevolution method, namely, DNN-COCA, for deep neural network architecture generation. Specifically, their model divided a population into two sub-populations $P_{1}$ and $P_{2}$ and applied a different search strategy in each sub-population. The sub-population $P_{1}$ employed a competitive co-evolution search method, whereas $P_{2}$ adopted a cooperative search strategy. To maintain search diversity, the work introduced an interpopulation migration strategy that migrated individuals between $P_{1}$ and $P_{2}$. A table of the best individuals from both populations was maintained and used to generate offspring solutions. The model achieved an accuracy rate of 98.7% on the MNIST dataset by evolving a total number of 5–7 layers within a CNN. Moreover, such constrained settings limit their model performance.

Calisto and Lai-Yuen [44] employed a multi-objective evolutionary algorithm based on decomposition (MOEA/D) [45] for the automatic construction of an ensemble of 2D and 3D residual models for medical image segmentation. A 2D CNN model extracted in-plane intra-slice information, while a 3D CNN model exploited volumetric inter-slice information. The work employed a multi-objective cost function that minimized the error rate and the number of model parameters simultaneously. The algorithm optimized the number of residual blocks, the number of filters of the first residual block, the kernel size of convolutional layers within each block, the activation function, dropout and learning rates, for both 2D and 3D models. Evaluated using the prostate segmentation task in the PROMISE12 Grand Challenge [46], the model achieved an impressive pixel-wise classification accuracy rate of 89.29%, ranked among the top 10 results for the challenge at the time of publication. In addition, GA and grey wolf optimizer (GWO) were adopted for optimal hyper-parameter identification and network topology optimization in the LSTM fully convolutional network (LSTM-FCN) and convolutional neural network-long short-term memory (CNN-LSTM) networks by Ortego et al. [47] and Xie et al. [48], respectively, for time-series prediction, with respect to diverse classification and regression problems.

## 3. The Proposed PSO-Based Deep Architecture Generation

In this research study, we propose a PSO-based deep architecture generation method, namely resPsoCnn, to devise deep networks with residual connections. The novel aspects of the proposed resPsoCnn model include (1) a new residual group-based encoding scheme and (2) a new search mechanism guided by neighboring and global promising solutions for deep architecture search. Specifically, the proposed encoding scheme is capable of representing deep CNN models comprising residual connections. The important settings, such as the number of groups and residual blocks, the kernel size and number of filters for each residual block, as well as pooling layer choices for each group, are optimized using the proposed resPsoCnn algorithm. Moreover, the search process led by the swarm leader and non-uniformly randomly selected neighboring promising solutions at the fine-grained and global levels illustrates a better balance between diversification and exploitation. Thus, it produces a rich assortment of residual architectures with great diversity. We introduce each key element of resPsoCnn in the following sub-sections.

### 3.1. Encoding Strategy and Initialization

The proposed encoding strategy stores multi-dimensional swarm position information for representing CNN model architecture configurations. At the start of the optimization process, a swarm containing a fixed number of particles is initialized with random particle positions constrained by predetermined search ranges. The following elements summarize the CNN architecture settings encoded within the proposed encoding strategy:

- A model contains at least one group. We optimize the number of groups between 1 and $g_{max}$.
- A group contains at least one residual block. The number of blocks the model can contain during initialization is set between 1 and $b_{max}$. We optimize the number of residual blocks in each group.
- All blocks within a group share the same number of channels for compatibility. We optimize the number of channels used by a group between $out_{min}$ and $out_{max}$.
- A group contains an optional pooling layer, which can be of the following types: max pooling, average pooling or no pooling. We optimize the pooling type by dividing a search range between 0 and 1 into three regions and attribute a pooling type to each region.
- A block contains a stack of convolutions layers, performing the same convolutions, i.e., the appropriate padding is used to ensure the dimensions of the output match those of the input volume. The degree of padding depends on the kernel size. The kernel size of a convolutional layer is optimized on a block-by-block basis between $k_{min}$ and $k_{max}$. This is necessary, as the kernel size controls the receptive field, which, in turn, controls the visibility degree of an image with respect to one filter, at one time [1].

The parameters optimized by the proposed PSO algorithm, including their search ranges, are summarized in Table 1.

### 3.2. Decoding Strategy

**Figure 2:**
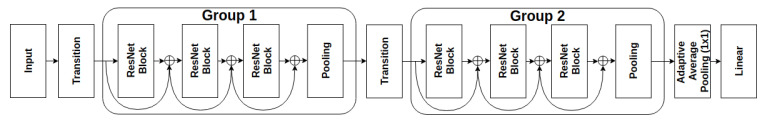
An example decoded network where the model configurations, i.e., the number of groups, the number of blocks per group and the contents of each group (e.g., the kernel size of each ResNet block, the number of channels and the pooling type for each group), are embedded in the encoding process.

The position information encoded within a particle is decoded to construct a valid CNN model architecture. As an example, a high-level overview of a constructed CNN model after the decoding process is visualized in Figure 2.

**Figure 3:**
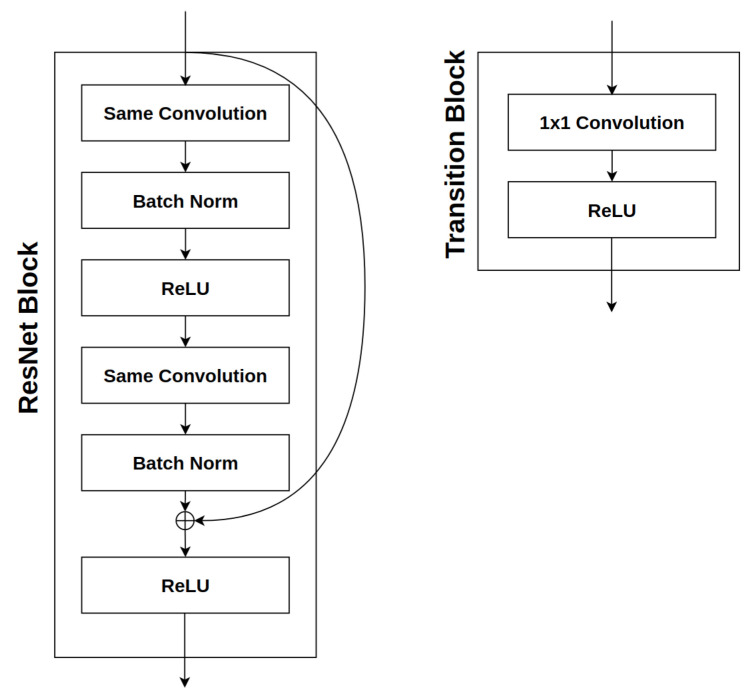
The structures of a ResNet block (**left**) and a transition block (**right**).

Skip connections require that the number of output channels from the previous layer matches that of the current layer, so that an add operation can be performed. A transition layer that precedes each group is used to either increase or decrease the number of output channels. Such a process ensures that the dimension of the input fed into a group matches the expected dimension of the group. A transition block comprises a 1 × 1 convolution and an ReLU activation function. A ResNet block comprises two stacked sets of a single convolutional layer, followed by a batch normalization layer and an ReLU layer. Both the transition block and the ResNet block are visualized in Figure 3.

In addition, the final group of a model is followed by an adaptive average pooling layer and a linear layer. The linear layer performs the final image classification with the number of neurons set as the number of target classes in the dataset.

### 3.3. The Optimization Strategy

The search of optimal architectures is conducted by optimizing important hyperparameters such as the kernel size and model depth. A list of the hyperparameters to be optimized is provided in Table 1. The optimization process is outlined in the following steps: In step 1, we calculate the particle differences with respect to the global and neighboring best solutions, as indicated in Section 3.4. In step 2, we calculate a new velocity based on the differences between the current particle position and the global and neighboring best solutions, as indicated in Section 3.5. In step 3, the position updating is performed by applying the new velocity to the current particle, as indicated in Section 3.6. The fitness of a new particle is subsequently evaluated using a fitness function, as described in Section 3.7. The above search process iterates until the termination criterion is fulfilled.

To benefit the discussion in the subsequent sub-sections, we define the following notations in Table 2. Specifically, they represents particle encoded information, such as the kernel size *k* of a particular block *b*, within the group *g* of a particle position *X*.

### 3.4. Particle Difference Calculation

During the optimization process, the generation of new particle velocity is an important step. The new velocity guides the particle movement by considering the differences between the best known positions, namely, $g_{best}$ and $p_{best}$, and the current individual. We adopt a velocity update rule for guiding particle search. It requires a mechanism for calculating the difference between a pair of particles. We propose a new method for particle difference calculation. At a high level, the difference of $X_{2}$ with respect to $X_{1}$ is calculated as $X_{1}-X_{2}$. This means that the resulting position difference could be negative or positive, based on the guidance of the global or neighboring promising solutions. If the difference is negative/positive, it means to update the particle with a smaller/larger network configuration setting than the current one. Moreover, owing to the search range constraints of hyperparameter settings provided in Section 4.2, it is not possible for an update to result in an out-of-boundary network configuration in our experiments. We introduce the proposed particle difference calculation method, as well as the new velocity and position updating formulae in the following sub-sections.

#### 3.4.1. Particle Difference Calculation between Groups with Respect to the Number of Channels $c_{out}$

We calculate the particle difference on a group-by-group basis. A group maintains a record of the number of output channels $c_{out}$ that each block within the group should use. Therefore, the difference with respect to $c_{out}$ between particles $X_{1}$ and $X_{2}$ is calculated by subtracting the current setting of $c_{out}$ with respect to particle $X_{2}$ from that of particle $X_{1}$ on a group-by-group basis to return $\Delta c_{out}$ for each group.

#### 3.4.2. Particle Difference Calculation between Groups with Respect to the Number of Blocks

**Figure 4:**
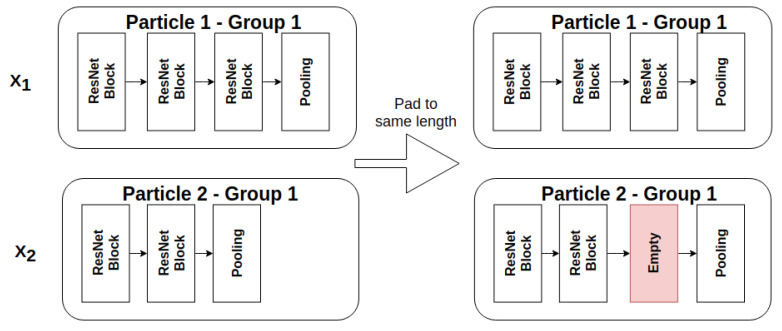
Groups from particles $X_{1}$ and $X_{2}$ which are temporarily padded to the same length in preparation for the particle difference calculation.

Groups vary with respect to the number of ResNet blocks they contain. We introduce a strategy to temporarily pad the groups of both particles to the same length by adding empty blocks to the one with a smaller number of blocks, as shown in Figure 4. The location of the empty block is used to decide where a block should be added or removed, as explained in Section 3.5.

#### 3.4.3. Particle Difference Calculation with Respect to the Block Kernel Size *k*

**Figure 5:**
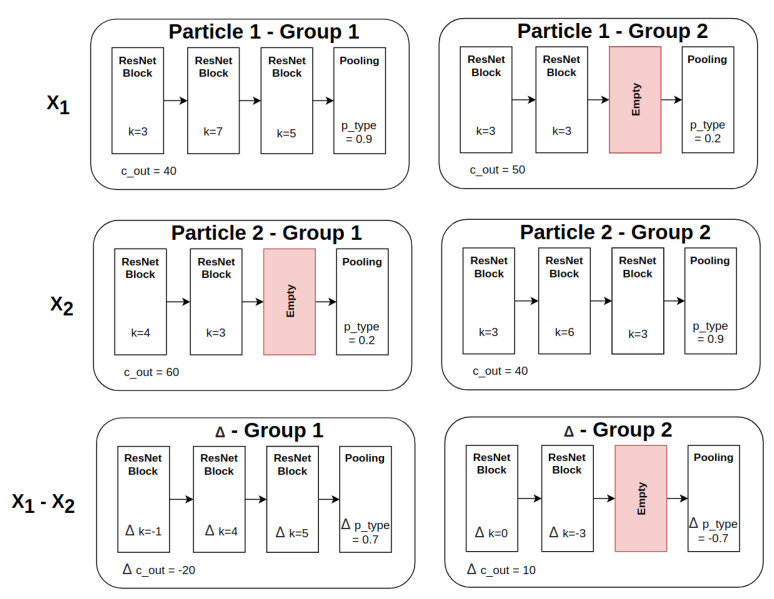
An example particle difference computation for $X_{1}-X_{2}$.

We compute the difference between two blocks with respect to the kernel size *k* by calculating $\Delta k=k_{\left(X_{1}g_{n}b_{m}\right)}-k_{\left(X_{2}g_{n}b_{m}\right)}$. Two special cases exist, i.e., (1) if the block from $X_{1}$ is an empty block as a result of the group padding described in Section 3.4.2, the output is also empty. Conversely, (2) if the block from $X_{2}$ is an empty block then the difference for the *m*th block is set as $\Delta k=k_{\left(X_{1}g_{n}b_{m}\right)}$, as shown in Figure 5.

#### 3.4.4. Particle Difference Calculation with Respect to the Pooling Type $p_{type}$

We compute the particle difference between two pooling layers with respect to the kernel pooling type $p_{type}$ as follows: We subtract the pooling type from the respective group of $X_{2}$ from that of $X_{1}$, in order to calculate $\Delta p_{type}$.

### 3.5. Velocity Calculation

Within a PSO algorithm, the velocity $V_{i}$ of particle *i* is calculated by computing the differences of particle position $X_{i}$ with respect to the global best solution $g_{best}$ and its personal best solution $p_{best}$. We propose a novel mechanism of velocity calculation, with two new features: (1) calculating velocity based on $g_{best}$ and a randomly selected neighboring best solution, namely, $n_{best}$, and (2) allowing velocity to be calculated based on the aforementioned encoding scheme. The hypothesis for using $n_{best}$ instead of $p_{best}$ within the proposed velocity calculation is that we intend to increase social communications and learning between neighboring particles. The adoption of randomly selected neighboring elite solutions is able to add a degree of randomness and encourage global exploration before converging toward the swarm leader.

The neighboring best position $n_{best}$ is a non-uniformly randomly selected particle from the swarm. We implement random selection using the python numpy [49] function random.choice(). The random.choice() function accepts a probability array containing values between 0 and 1. To compute the probability array, we use the losses of the entire swarm from the previous iteration $loss^{t-1}$ as the reference. Note that losses closer to 0 indicate better particle positions and we want to favor such particles with higher probabilities. To achieve this, we inverse the losses and scale them between 0 and 1 for probability array construction. A higher probability score for index *i* indicates a higher chance for particle *i* to be selected as $n_{best}$.

From the observations of the experiments, the selected neighboring promising solution in each iteration is more likely to be one of the top solutions in the swarm. Such an operation not only adds randomness to the guiding signals in the search process, but also ensures that the swarm is more likely to be guided towards optimal regions.

**Figure 6:**
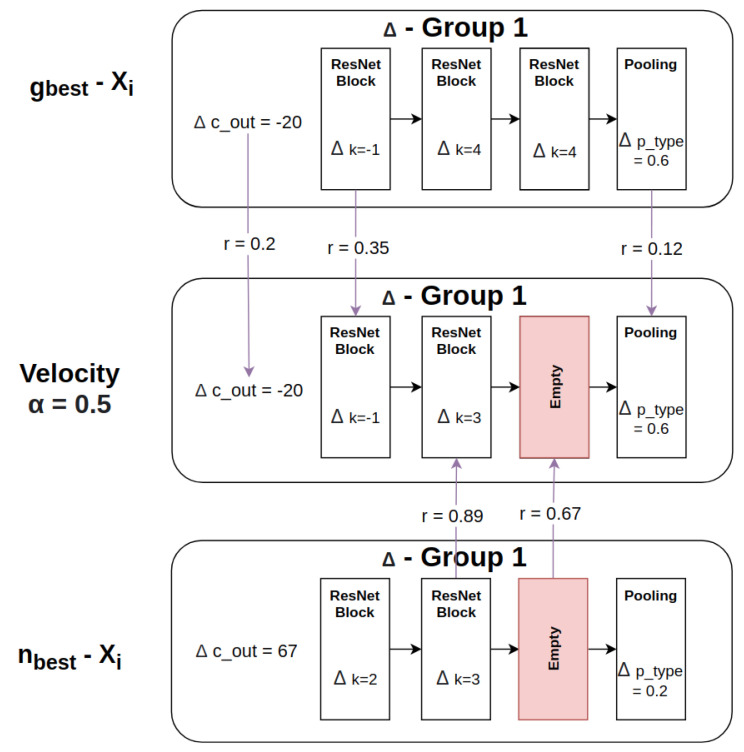
An example of velocity calculation between the selection of $n_{best}-X_{i}$ and $g_{best}-X_{i}$.

After determining $n_{best}$, we compute the difference between $n_{best}$ and the current particle *i*, i.e., $n_{best}-X_{i}$, as well as the difference between $g_{best}$ and the particle *i*, i.e., $g_{best}-X_{i}$. Then, we iterate over each block and the pooling layer. For every block, we generate a random number *r* between 0 and 1. We compare the random number *r* against a threshold α. If r≤α, the difference of $g_{best}-X_{i}$ is selected, otherwise the difference of $n_{best}-X_{i}$ is adopted, as shown in Figure 6. A similar process is also conducted for pooling-layer generation.

The above velocity updating operation is conducted in three steps, i.e., (1) a selection is made for $\Delta c_{out}$ for each group, (2) a selection is made for Δk for each ResNet block within each group and (3) a selection is made for $\Delta p_{type}$ for each group. The velocity generation procedure is diversified by introducing randomness into the selection criteria in each step, as depicted in Equation (3). Owing to the guidance of multiple neighboring and global elite solutions in velocity updating in block and group levels, the search process is equipped with better diversity and capabilities in overcoming local optimum traps.

(3) $$velocitySelection_{PerGroupAndBlock}=\left\{\begin{array}{cc} g_{best}-X_{i} & \mathrm{if}\;r\leq \alpha \\ n_{best}-X_{i} & \mathrm{otherwise} \end{array}\right.$$

### 3.6. Position Updating

Once the new velocity $V_{i}$ has been calculated, the position of particle $X_{i}^{t+1}$ can be updated by adding the weighted velocity to the current particle position $X_{i}^{t}$. The weighting factor β controls the degree at which the new velocity is added to the current position. Higher values of β result in larger degrees of movement, whereby smaller values of β encourage a granular search of intermediate positions. We set β=0.5 in our experiments to balance between exploration and exploitation. The modified position-updating formula is provided in Equation (4).

Two special cases exist when applying velocity. (1) If an empty block within velocity $V_{i}$ corresponds to a non-empty block in particle position $X_{i}$, the block is removed from the particle position $X_{i}$. (2) If a non-empty block within $V_{i}$ corresponds to an empty block in particle position $X_{i}$, the block from velocity $V_{i}$ is copied into particle position $X_{i}$. In this way, a positive value of *k* is ensured based on the definitions of both special cases.

(4) $$X_{i}^{t+1}=\beta V_{i}+X_{i}^{t}$$

### 3.7. Fitness Evaluation

Each particle *i* within a swarm is evaluated by decoding the particle position *X* to construct a new CNN model. We trained the new model on a training set using the Adam optimizer [50] with a learning rate of 0.001 for 1 epoch. Such training settings were adopted from existing studies, i.e., sosCNN [14] and psoCNN [15], for the purpose of direct comparison. The average cross-entropy loss during training was used as the fitness score. The overall objective of the optimization process is to minimize the fitness scores by improving the particle positions over a number of iterations.

We employed the proposed encoding scheme and search operations guided by multiple elite solutions for optimizing deep architectures with residual connections. A comprehensive evaluation was conducted, which is elaborated in detail in the next section.

## 4. Experimental Studies

### 4.1. Datasets

We evaluated our proposed model using six well-known benchmark datasets for direct comparison against closely related studies in similar settings. The adopted datasets were Rectangles-I, MNIST and four MNIST variant datasets. In comparison with the MNIST dataset, the four MNIST variant datasets are more challenging owing to the associated transformations such as rotations, addition of backgrounds, as well as other distracting factors.

Table 3 provides a summary of the experimental datasets including the official training and test split sample sizes.

### 4.2. Parameter Settings

We adopt the settings shown in Table 4 in our experiments. Specifically, we set the maximum number of groups $g_{max}$ to 2 owing to the selected input image sizes (28 × 28) provided by the datasets. As each group could potentially contain a pooling layer and each pooling layer halves the output dimension size, adding more groups could negatively impact performance by reducing the output dimension too aggressively. For datasets containing larger input sizes, larger values for $g_{max}$ could be selected.

We selected a pooling layer type of a group based on the current value of $p_{type}$, which ranged between 0 and 1. The pooling operation selected based on the current value of $p_{type}$ is explained in Equation (5).

(5) $$pooling=\left\{\begin{array}{cc} NoPooling & \mathrm{if}\;p_{type}\leq 0.33,\\ AvePooling & \mathrm{if}\;p_{type}>0.33\;\&\;\;p_{type}\leq 0.66,\\ MaxPooling & \mathrm{otherwise} \end{array}\right.$$

### 4.3. Benchmark Models

To test model efficiency, hand-crafted networks such as LeNet [3] and several deep architecture generation methods, i.e., IPPSO [21], MBO-ABCFE [35], GeNet [16], DNN-COCA [43], psoCNN [15] and sosCNN [14], were employed for performance comparison.

IPPSO [21] is a PSO-based method for designing CNN architectures. It employs a test methodology consisting of 20 particles over 10 iterations. The search process of IPPSO is constrained to a maximum of 9 convolutional layers with kernel sizes ranging between 1 and 8 and 3 fully connected layers.

MBO-ABCFE [35] is a variant of the MBO algorithm [34], which incorporates the search mechanisms of ABC [37] and FA [38] to increase exploration and exploitation of MBO, respectively. The experiment was conducted with a population size of 50, over 50 iterations. Their optimized hyperparameters included the number of convolutional layers between 1 and 4, kernel size between 2 and 9 and the number of filters as either of the following: 8, 16, 32, 64, 128, 256, 512 and 1024. Their model also optimized the activation function type as either ReLU or a linear activation function and batch size as 25, 50, 100 or 200.

GeNet [16] is a GA-based approach for deep network generation. GeNet adopts a block-based architecture design with a fixed kernel size and a fixed number of filters per block. The model adopts a search strategy that optimizes the connections between network blocks, forming nonlinear block connection patterns. The model was evaluated using the MNIST dataset and achieved an error rate of 0.34%, with a population size of 20 individuals over 50 iterations.

DNN-COCA [43] employs a multi-population strategy which adopts competitive and cooperative neuroevolution methods for the search of optimal architectures. The model was evaluated using the MNIST dataset. It generated CNNs of depths between 5 and 7 layers and achieved an accuracy rate of 98.7%.

psoCNN [15] is another PSO-based deep architecture generation method. It introduces a flexible encoding mechanism which describes a CNN model as an array. Each element within the array denotes a type of layer. The possible layer types comprise convolutions layers with kernel sizes between 3 × 3 and 7 × 7 inclusive and a maximum of 256 channels, as well as pooling layers and fully connected layers. Based on a selection criterion, the position of a particle within a swarm size of 20 is optimized over 10 iterations on a layer-by-layer basis. The new layer type and its settings are determined by choosing either from the global or personal best solution.

Finally, sosCNN [14] is an SOS [32]-based deep architecture generation method. It uses a swarm of 20 individuals, optimized over 10 iterations. The hyperparameters selected for optimization are the layer types, i.e., convolution layers with kernel sizes between 3 × 3 and 7 × 7 and up to 256 filters, pooling layers or fully connected layers. The maximum number of layers is limited to a total of 20.

### 4.4. Results

#### 4.4.1. Performance Comparison with Existing Studies

We employed the following settings in our experimental studies: a population of 20 and a maximum number of iterations of 10—as used in existing studies, e.g., IPPSO [21], psoCNN [15] and sosCNN [14]. During the search stage, we train each devised network with 1 epoch. The best model identified at the end of the search phase was trained for 100 epochs. A set of 10 trials was conducted.

In Table 5, we present our results against those of the aforementioned benchmark models, i.e., hand-crafted models LeNet-1, LeNet-4 and LeNet-5 [3], as well as evolutionary methods, i.e., IPPSO [21], MBO-ABCFE [35], GeNet [16], DNN-COCA [43], psoCNN [15] and sosCNN [14]. All reported results pertaining to the benchmark models are taken from their respective publications, to ensure a fair comparison.

In the last two rows of Table 5, we present the best and mean classification error rates over 10 runs achieved by the proposed model, resPsoCnn. The remaining rows are the best and mean error rates (where available) reported by the compared methods in their original studies. The best results are highlighted in bold for a given dataset.

As indicated by the reduction in the error rates reported across all the benchmark datasets, in comparison with the baseline methods, the proposed model showed better performances in most test cases. In addition, sosCNN was the best performing compared method across all datasets. In Table 6, we compare the error rates of resPsoCnn against those of sosCNN [14], to clearly indicate performance improvement.

MNIST represents a relatively simple handwritten digit classification problem with a small margin available for improvement. For MNIST, as indicated in Table 6, sosCNN reported a mean error rate of 0.40%. In comparison, our model, resPsoCnn, achieved the lowest mean error rate of 0.33%, with an improvement of 0.07% over sosCNN.

For the MNIST-RD dataset, sosCNN obtained a mean error rate of 3.78% and the top-1 error rate of 3.01%. resPsoCnn showed a mean error rate of 3.02% and a top-1 error rate of 2.67%, with improvements of 0.76% and 0.34% over sosCNN, respectively.

For the MNIST-RB dataset, sosCNN achieved a mean error rate of 1.89%. resPsoCnn obtained a mean error rate of 1.76%, with an improvement of 0.13% over sosCNN.

resPsoCnn achieved a mean error rate of 1.90% with respect to the MNIST-BI dataset, with an improvement of 0.08% over the mean error rate of 1.98% obtained by sosCNN.

The MNIST-RD+BI dataset depicted a more challenging classification problem due to the composition of rotated MNIST digits and background images. For this dataset, sosCNN obtained a mean error rate of 13.61% and a top-1 error rate of 10.65%. resPsoCnn showed a mean error rate of 9.27% and a top-1 error rate of 8.76%, with improvements of 4.34% and 1.89% over sosCNN, respectively.

For the Rectangles-I dataset, sosCNN reported a mean error rate of 2.37% and a top-1 error rate of 1.57%. resPsoCnn depicted a mean error rate of 1.47% and a top-1 error rate of 1.19%, with improvements of 0.38% and 0.90% over sosCNN, respectively.

#### 4.4.2. Evaluation of the Proposed Encoding and Search Strategies

To further demonstrate the contributions to the overall results gained from the proposed encoding scheme and multiple leader-guided search strategy, we performed additional experiments to isolate the two proposals.

As mentioned earlier, we refer to our overall model as resPsoCnn. In addition, we denote the version using our proposed encoding strategy isolated from the proposed search strategy as resPsoCnn-PB-GB. Moreover, resPsoCnn-PB-GB uses the original PSO operation based on the personal and global best solutions to guide the search process. The purpose of providing two sets of results is to demonstrate the contribution of each strategy in isolation from the other.

In Table 7, rows 1 and 2 indicate the top-1 and mean error rates of the best performing compared model, sosCNN. Rows 3 and 4 show the results of resPsoCnn-PB-GB, where our encoding strategy in combination with the original PSO operation was used for architecture search.

Rows 5 and 6 indicate the performance of the overall proposed model, resPsoCnn, where both proposed encoding and search strategies are combined. Specifically, the architecture search in resPsoCnn is led by the swarm leader and a non-uniformly selected neighboring best solution.

For the MNIST dataset, the mean and top-1 error rates of sosCNN were 0.40% and 0.30%, respectively. resPsoCnn-PB-GB, with our encoding strategy alone, obtained identical results in terms of the mean and top-1 error rates. When both proposed strategies were combined, resPsoCnn achieved a mean error rate of 0.33%, with an improvement of 0.07% over sosCNN.

For the MNIST-RD dataset, resPsoCnn-PB-GB achieved a mean error rate of 3.23% and a top-1 error rate of 2.84%, with improvements of 0.55% and 0.17% over those of sosCNN, respectively. Furthermore, when the proposed encoding and search strategies were combined, resPsoCnn illustrated more significant improvements, of 0.76% and 0.34%, in terms of the mean and top-1 error rates, respectively, over sosCNN.

With respect to the MNIST-RB dataset, the mean error rate reported by sosCNN is 1.89%. The mean error rates of resPsoCnn-PB-GB and resPsoCnn were both 1.76%, i.e., an improvement of 0.13% in respect to sosCNN. Furthermore, resPsoCnn-PB-GB performed better than resPsoCnn with respect to the top-1 error rate, with an error difference of 0.19% between the two models.

For the MNIST-BI dataset, the mean error rate reported by sosCNN is 1.98%. The mean error rate of resPsoCnn-PB-GB was 2.02%. When combining both proposed strategies, the mean error rate of resPsoCnn increased to 1.90%, with an improvement of 0.08% over sosCNN. The best top-1 error rate, 1.68%, was achieved by sosCNN, whereas resPsoCnn achieved a top-1 error rate of 1.74%.

For the MNIST-RD+BI dataset, the mean and the top-1 error rates of resPsoCnn-PB-GB were 9.74% and 9.20%, with improvements of 3.87% and 1.45% over sosCNN, respectively. Furthermore, the mean and the top-1 error rates of resPsoCnn were 9.27% and 8.76%, with improvements of 4.34% and 1.89% over sosCNN, respectively.

With respect to the Rectangles-I dataset, resPsoCnn-PB-GB achieved the lowest top-1 error rate of 0.89%, with an improvement of 0.68% over sosCNN. resPsoCnn achieved the lowest mean error rate of 1.47%, with an improvement of 0.9% over sosCNN.

In summary, resPsoCnn-PB-GB, with the proposed encoding strategy, only illustrated performance enhancement over sosCNN for the MNIST-RD, MNIST-RB, MNIST-RD+BI and Rectangles-I datasets. resPsoCnn, combining both the proposed encoding and search strategies, resulted in a further improvement of performance for all the six datasets. Moreover, since resPsoCnn with the proposed search mechanism outperformed resPsoCnn-PB-GB with the original PSO operation, the results further indicate the effectiveness of the proposed movement strategy.

### 4.5. Theoretical Justification

In this research study, firstly, we propose a new encoding scheme capable of representing deep CNN architectures comprising residual blocks. Secondly, we propose a new search strategy that updates the particle position based on the swarm leader and a non-uniformly selected neighboring solution capable of overcoming stagnation.

**Figure 7:**
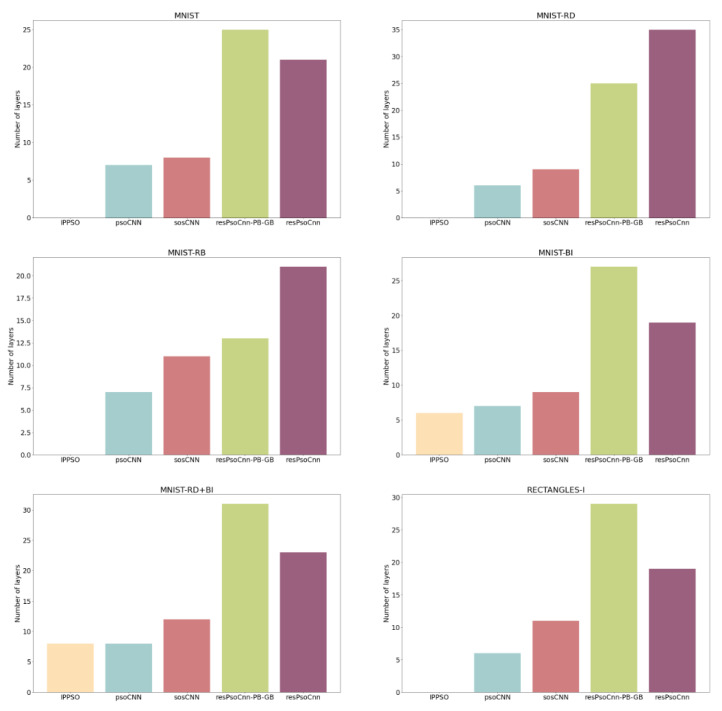
A comparison of model depths between the networks devised by the benchmark models and our methods, resPsoCnn-PB-GB and resPsoCnn, across all datasets.

In Figure 7, we compare the depths of the best networks produced by resPsoCnn-PB-GB and resPsoCnn against those of the benchmark models of IPPSO, psoCNN and sosCNN. The experimental results indicate that resPsoCnn-PB-GB and resPsoCnn are capable of producing deeper network architectures, as indicated by greater network depths across all datasets, owing to the introduction of skip connections. On the contrary, the compared models tend to produce shallower networks, i.e., their devised networks are limited in terms of the maximum depths due to vanishing gradients. Specifically, the vanishing gradient problem impacts a model’s ability to learn. In such a situation, backpropagation is unable to adjust weights as the gradients become 0; therefore, learning stops. Skip connections minimize the vanishing gradient problem; therefore, they allow resPsoCnn-PB-GB and resPsoCnn to form deeper networks. Moreover, the empirical results in Table 5 and Table 7 indicate the advantage of constructing deeper models pertaining to performance enhancement. This is ascertained by the reduction in the mean error rates across all datasets for both resPsoCnn-PB-GB and resPsoCnn in respect to the compared methods.

Furthermore, the results in Table 7 indicate that resPsoCnn outperformed resPsoCnn-PB-GB in terms of the mean error rates across all datasets. This confirms the benefits of combining the proposed encoding scheme and search strategy based on the neighboring and global best solutions. The empirical results indicate that the proposed search mechanism improves search diversity and prevents the search from being trapped in local optima. This is due to the randomness introduced by the non-uniform neighboring solution selection mechanism explained in Section 3.5.

Table 8 illustrates the structures of the best models generated by resPsoCnn-PB-GB, while Table 9 presents the topologies of the best models devised by resPsoCnn. TB indicates a transitional block, while RB indicates a residual block, as shown in Figure 3. FC denotes the final fully connected layer (i.e., the linear layer) of the model, as indicated in Figure 2. The networks constructed by resPsoCnn are more diversified in the selected pooling layers. As indicated in Table 8, all pooling types selected by resPsoCnn-PB-GB are average pooling with the exception of MNIST-RD, which selects no pooling for the first group. In contrast, as illustrated in Table 9, resPsoCnn selects max-pooling in the second groups for MNIST-RB and MNIST-BI and no pooling for any groups for Rectangles-I. This indicates that resPsoCnn has better search diversity by selecting from the global and neighboring best solutions with respect to block and group configuration generation. It shows better capabilities in escaping from local optimum traps in relation to the pooling layer selection. Moreover, the improvement in search exploration has, in turn, resulted in the reduction in error rates yielded by resPsoCnn in comparison with resPsoCnn-PB-GB, as indicated in Table 7.

## 5. Conclusions

In this research study, we propose an automatic approach for optimizing architectures of CNNs. We make attempts to overcome two weaknesses in the existing studies. Firstly, existing CNN architecture generation techniques do not consider skip connections. The importance of skip connections is that they help overcome vanishing gradient problems. Without skip connections, the networks devised by existing studies are limited in terms of model depths. Secondly, existing studies conduct optimization of residual networks but reduce search space by eliminating fine-grained network setting choices. Specifically, they purely focus on optimizing the model depth and width, while using fixed kernel sizes and pooling types. This may limit the diversity of the generated networks. In addition, existing studies are more inclined to adopt a search process guided by the original PSO operation, making them vulnerable to local optimum traps.

Therefore, to address the above drawbacks, we propose (1) a novel residual group-based encoding strategy capable of representing network configurations with residual connections to better tackle vanishing gradient problems and (2) a search mechanism guided by neighboring and global best solutions to escalate social communication to avoid stagnation.

With respect to the proposed residual group-based encoding strategy, Figure 7 indicates that our models are capable of generating deeper architectures than those yielded by related studies, such as IPPSO, psoCNN and sosCNN, all of which do not exploit residual connections. In addition, our devised networks show better capabilities in tackling vanishing gradients owing to the adoption of skip connections. As an example, resPsoCnn-PB-GB, with the proposed encoding strategy, only showed performance improvement over sosCNN for the MNIST-RD, MNIST-RB, MNIST-RD+BI and Rectangles-I datasets, as indicated in Table 7.

With respect to the proposed movement strategy, the empirical results in Table 5 indicate that our search process guided by the neighboring and global best solutions resulted in more accurate networks, in comparison with those yielded by the related studies (e.g., IPPSO, psoCNN and sosCNN), across all six datasets. In addition, as illustrated in Table 7, since resPsoCnn, with the proposed movement mechanism, outperformed resPsoCnn-PB-GB with the original PSO operation in terms of mean accuracy rates, the results further indicate the effectiveness of the proposed search strategy.

Furthermore, when combining our movement and encoding strategies, we observed a further performance improvement in comparison with those of related studies, as shown in Table 5. In particular, resPsoCnn achieved the most significant improvement of 4.43% over the best baseline method, sosCNN, on the MNIST-RD+BI dataset.

In future work, we will explore adding dense connections and optimizing the connection types, i.e., selecting between skip and dense connections, for the generation of new model structures. Moreover, in order to ensure a fair comparison with related studies, we do not apply data augmentation techniques in this research work. For future experiments, we will explore data augmentation as it is a useful technique for avoiding the overfitting of deeper networks and it provides better generalization capabilities to enhance performance. Besides that, we aim to employ an adaptive weighting factor, β, to adaptively control the strength of new velocity on position updating. An exponential moving average of the most recent velocities will also be considered for new velocity generation to smooth the particle movement. Finally, we aim to evaluate the proposed model for generating deep architectures with respect to other complex computer vision tasks [55], such as object detection, semantic segmentation, video description and visual question generation.

## Figures and Tables

**Table 1:** The optimized network parameters and their corresponding search ranges. The settings of the search ranges used in our experiments are provided in Section 4.

Domain	Parameter	Range
Model	Number of groups	from 1 to $g_{max}$
Group	Number of residual blocks	from 1 to $b_{max}$
Group	The number of channels $c_{out}$ for all blocks in a group	from $out_{min}$ to $out_{max}$
Convolution	Kernel size *k*	from $k_{min}$ to $k_{max}$
Pooling	Pooling type $p_{type}$	from 0 to 1

**Table 2:** A summary of the notations.

Notation	Description
$X_{i}$	The position *X* of the *i*th particle in the swarm
$g_{n}$	The *n*th group
$b_{m}$	The *m*th block
$k_{\left(X_{i}g_{n}b_{m}\right)}$	Kernel size for the *m*th block of the *n*th group of the *i*th particle in position *X*

**Table 3:** A summary of the datasets used in our experiments. All datasets have an input size of 28 × 28 × 1 [51].

Dataset	Description	Classes	Train/Test Samples
MNIST [3,52]	Handwritten digits	10	60,000/10,000
MNIST-RD [53,54]	Rotated MNIST digits	10	12,000/50,000
MNIST-RB [53,54]	MNIST digits with random background noise	10	12,000/50,000
MNIST-BI [53,54]	MNIST digits with background images	10	12,000/50,000
MNIST-RD+BI [53,54]	Rotated MNIST digits with background images	10	12,000/50,000
Rectangles-I [53,54]	Rectangle border shapes with background images	2	12,000/50,000

**Table 4:** Algorithm settings and the search space in our experiments. We adopt the settings to closely match those of existing studies [15,51], to ensure a fair comparison.

Name	Description	Value Used
$k_{min}$	Minimum kernel size	3
$k_{max}$	Maximum kernel size	7
$out_{min}$	Minimum number of channels	16
$out_{max}$	Maximum number of channels	256
$b_{max}$	Maximum number of blocks	15
$g_{max}$	Maximum number of groups	2
α	Layer selection boundary threshold	0.5
β	Velocity weighting factor	0.5

**Table 5:** Experimental results of the proposed model, resPsoCnn and those of the compared methods extracted from their original studies. The best results are highlighted in bold for a given dataset.

Model	MNIST	MNIST-RD	MNIST-RB	MNIST-BI	MNIST-RD+BI	Rectangles-I
Hand-crafted architectures						
LeNet-1 [3]	1.70%	19.3%	7.50%	9.80%	40.06%	16.92%
LeNet-4 [3]	1.10%	11.79%	6.18%	8.96%	33.83%	16.09%
LeNet-5 [3]	0.95%	11.10%	5.99%	8.70%	34.64%	12.48%
Evolutionary algorithms for architecture generation						
IPPSO (best) [21]	1.13%	-	-	-	33%	-
IPPSO (mean) [21]	1.21%	-	-	-	34.50%	-
MBO-ABCFE (best) [35]	0.34%	-	-	-	-	-
GeNET (best) [16]	0.34%	-	-	-	-	-
DNN-COCA (mean) [43]	1.30%	-	-	-	-	-
psoCNN (best) [15]	0.32%	3.58%	1.79%	1.90%	14.28%	2.22%
psoCNN (mean) [15]	0.44%	6.42%	2.53%	2.40%	20.98%	3.94%
sosCNN (best) [14]	**0.30**%	3.01%	1.49%	**1.68%**	10.65%	1.57%
sosCNN (mean) [14]	0.40%	3.78%	1.89%	1.98%	13.61%	2.37%
**resPsoCnn (best)**	0.31%	**2.67**%	1.70%	1.74%	**8.76**%	1.19%
**resPsoCnn (mean)**	**0.33**%	**3.02**%	**1.76**%	**1.90**%	**9.27**%	**1.47**%

**Table 6:** The best and mean error rates over 10 runs for the proposed method, resPsoCnn and the best baseline method, sosCNN [14], where a (−)/(+) symbol indicates that resPsoCnn performed better/worse than sosCNN.

Model	MNIST	MNIST-RD	MNIST-RB	MNIST-BI	MNIST-RD+BI	Rectangles-I
**resPsoCnn (best)**	0.31%	**2.67%**	1.70%	1.74%	**8.76%**	**1.19%**
**resPsoCnn (mean)**	**0.33%**	**3.02%**	**1.76%**	**1.90%**	**9.27%**	**1.47%**
sosCNN (best) [14]	**0.30**%	3.01%	**1.49%**	**1.68%**	10.65%	1.57%
sosCNN (mean) [14]	0.40%	3.78%	1.89%	1.98%	13.61%	2.37%
**error difference (best)**	0.01%(+)	**−0.34%(−)**	0.21%(+)	0.06%(+)	**−1.89%(−)**	**−0.38%(−)**
**error difference (mean)**	**−0.07%(−)**	**−0.76%(−)**	**−0.13%(−)**	**−0.08%(−)**	**−4.34%(−)**	**−0.90%(−)**

**Table 7:** Evaluation results of resPsoCnn (the proposed encoding scheme in combination with the proposed search strategy), resPsoCnn-PB-GB (the proposed encoding scheme in combination with the original PSO operation) and sosCNN.

Model	MNIST	MNIST-RD	MNIST-RB	MNIST-BI	MNIST-RD+BI	Rectangles-I
sosCNN (best) [14]	**0.30**%	3.01%	**1.49%**	**1.68%**	10.65%	1.57%
sosCNN (mean) [14]	0.40%	3.78%	1.89%	1.98%	13.61%	2.37%
**resPsoCnn-PB-GB (best)**	**0.30**%	2.84%	1.51%	1.79%	9.20%	**0.89**%
**resPsoCnn-PB-GB (mean)**	0.40%	3.23%	**1.76**%	2.02%	9.74%	1.66%
**resPsoCnn (best)**	0.31%(+)	**2.67%(−)**	1.70%(+)	1.74%(+)	**8.76%(−)**	1.19%(+)
**resPsoCnn (mean)**	**0.33%(−)**	**3.02%(−)**	**1.76%(−)**	**1.90%(−)**	**9.27%(−)**	**1.47%(−)**

**Table 8:** The identified best models for all benchmark datasets using resPsoCnn-PB-GB. TB indicates a transitional block which contains a single 1 × 1 convolutional layer and RB indicates a ResNet block which contains two convolutions, as indicated in Figure 3. FC indicates a fully connected layer.

Dataset	Structure
MNIST [3,52]	TB($c_{in}=1$ $c_{out}=177$) + RB(177 × 4 × 4) + RB(177 × 4 × 4) + RB(177 × 6 × 6) + AveragePool + TB($c_{in}=177$ $c_{out}=175$) + RB(175 × 6 × 6) + RB(175 × 6 × 6) + RB(175 × 5 × 5) + RB(175 × 3 × 3) + AveragePool + FC
MNIST-RD [53,54]	TB($c_{in}=1$ $c_{out}=161$) + RB(161 × 5 × 5) + RB(161 × 7 × 7) + RB(161 × 6 × 6) + RB(161 × 6 × 6) + RB(161 × 4 × 4) + RB(161 × 5 × 5) + RB(161 × 7 × 7) + TB($c_{in}=161$ $c_{out}=115$) + RB(115 × 5 × 5) + RB(115 × 7 × 7) + RB(115 × 5 × 5) + RB(115 × 6 × 6) + RB(115 × 3 × 3) + RB(115 × 7 × 7) + RB(115 × 4 × 4) + AveragePool + FC
MNIST-RB [53,54]	TB($c_{in}=1$ $c_{out}=153$) + RB(153 × 4 × 4) + RB(153 × 6 × 6) + + RB(153 × 4 × 4) + RB(153 × 3 × 3) AveragePool + TB($c_{in}=153$ $c_{out}=183$) + RB(183 × 4 × 4) + RB(183 × 6 × 6) + RB(183 × 7 × 7) + AveragePool + FC
MNIST-BI [53,54]	TB($c_{in}=1$ $c_{out}=136$) + RB(136 × 4 × 4) + RB(136 × 3 × 3) + RB(136 × 5 × 5) + RB(136 × 3 × 3) + AveragePool + TB($c_{in}=136$ $c_{out}=136$) + RB(136 × 6 × 6) + RB(136 × 5 × 5) + RB(136 × 5 × 5) + RB(136 × 3 × 3) + RB(136 × 3 × 3) + RB(136 × 3 × 3) + AveragePool + FC
MNIST-RD+BI [53,54]	TB($c_{in}=1$ $c_{out}=150$) + RB(231 × 5 × 5) + RB(231 × 5 × 5) + RB(231 × 7 × 7) + RB(231 × 3 × 3) + AveragePool + TB($c_{in}=150$ $c_{out}=98$) + RB(120 × 4 × 4) + RB(120 × 6 × 6) + RB(120 × 6 × 6) + RB(120 × 5 × 5) + AveragePool + FC
RECTANGLES-I [53,54]	TB($c_{in}=1$ $c_{out}=195$) + RB(195 × 3 × 3) + RB(195 × 6 × 6) + RB(195 × 3 × 3) + AveragePool + TB($c_{in}=195$ $c_{out}=85$) + RB(85 × 7 × 7) + RB(85 × 5 × 5) + RB(85 × 3 × 3) + AveragePool + FC

**Table 9:** The identified best models for all benchmark datasets using resPsoCnn. TB indicates a transitional block which contains a single 1 × 1 convolutional layer and RB indicates a ResNet block which contains two convolutions, as indicated in Figure 3. FC indicates a fully connected layer.

Dataset	Structure
MNIST [3,52]	TB($c_{in}=1$ $c_{out}=176$) + RB(176 × 4 × 4) + RB(176 × 5 × 5) + RB(176 × 5 × 5) + RB(176 × 4 × 4) + RB(176 × 5 × 5) + RB(176 × 3 × 3) + AveragePool + TB($c_{in}=176$ $c_{out}=198$) + RB(198 × 5 × 5) + RB(198 × 6 × 6) + RB(198 × 4 × 4) + RB(198 × 4 × 4) + AveragePool + FC
MNIST-RD [53,54]	TB($c_{in}=1$ $c_{out}=184$) + RB(184 × 4 × 4) + RB(184 × 4 × 4) + RB(184 × 5 × 5) + RB(184 × 4 × 4) + RB(184 × 3 × 3) + RB(184 × 3 × 3) + AveragePool + TB($c_{in}=184$ $c_{out}=146$) + RB(146 × 4 × 4) + RB(146 × 4 × 4) + RB(146 × 5 × 5) + RB(146 × 3 × 3) + AveragePool + FC
MNIST-RB [53,54]	TB($c_{in}=1$ $c_{out}=216$) + RB(216 × 5 × 5) + RB(216 × 6 × 6) + AveragePool + TB($c_{in}=216$ $c_{out}=158$) + RB(158 × 7 × 7) + RB(158 × 4 × 4) + Ma × Pool + FC
MNIST-BI [53,54]	TB($c_{in}=1$ $c_{out}=188$) + RB(188 × 4 × 4) + RB(188 × 5 × 5) + RB(188 × 5 × 5) + RB(188 × 4 × 4) + RB(188 × 4 × 4) + RB(188 × 3 × 3) + RB(188 × 3 × 3) + AveragePool + TB($c_{in}=188$ $c_{out}=177$) + RB(177 × 5 × 5) + RB(177 × 3 × 3) + RB(177 × 3 × 3) + RB(177 × 3 × 3) + Ma × Pool + FC
MNIST-RD+BI [53,54]	TB($c_{in}=1$ $c_{out}=231$) + RB(231 × 4 × 4) + RB(231 × 5 × 5) + RB(231 × 5 × 5) + RB(231 × 4 × 4) + RB(231 × 3 × 3) + RB(231 × 4 × 4) + AveragePool + TB($c_{in}=231$ $c_{out}=120$) + RB(120 × 5 × 5) + RB(120 × 5 × 5) + RB(120 × 3 × 3) + RB(120 × 4 × 4) + RB(120 × 4 × 4) + RB(120 × 3 × 3) + RB(120 × 3 × 3) + AveragePool + FC
RECTANGLES-I [53,54]	TB($c_{in}=1$ $c_{out}=71$) + RB(71 × 4 × 4) + RB(71 × 3 × 3) + RB(71 × 7 × 7) + RB(71 × 6 × 6) + RB(71 × 6 × 6) + RB(71 × 6 × 6) + TB($c_{in}=71$ $c_{out}=21$) + RB(21 × 5 × 5) + RB(21 × 4 × 4) + RB(21 × 6 × 6) + RB(21 × 6 × 6) + RB(21 × 7 × 7) + RB(21 × 4 × 4) + FC

## Data Availability

The data sets employed in this study are publicly available at the following sites, http://yann.lecun.com/exdb/mnist/ and http://www.iro.umontreal.ca/~lisa/icml2007data/.
